# Clinical development of CAR T cell therapy in China: 2020 update

**DOI:** 10.1038/s41423-020-00555-x

**Published:** 2020-09-30

**Authors:** Jianshu Wei, Yelei Guo, Yao Wang, Zhiqiang Wu, Jian Bo, Bin Zhang, Jun Zhu, Weidong Han

**Affiliations:** 1 Department of Bio-Therapeutic, The First Medical Center, Chinese PLA General Hospital, Beijing, 100853 China; 2 Department of Hematology, The First Medical Center, Chinese PLA General Hospital, Beijing, 100853 China; 3 Department of Hematopoietic Stem Cell Transplantation, The Fifth Medical Center, Chinese PLA General Hospital, Beijing, 100071 China; 4 Key Laboratory of Carcinogenesis and Translational Research, Departments of Lymphoma, Radiology and Nuclear Medicine, Peking University Cancer Hospital and Institute, Beijing, 100036 China

**Keywords:** chimeric antigen receptor, clinical trials, China, Cancer immunotherapy, Targeted therapies

## Abstract

Chimeric antigen receptor (CAR) T-cell therapy has achieved significant success in the treatment of hematological malignancies. In recent years, fast-growing CAR T clinical trials have actively explored their potential application scenarios. According to the data from the clinicaltrials.gov website, China became the country with the most registered CAR T trials in September 2017. As of June 30, 2020, the number of registered CAR T trials in China has reached 357. In addition, as many as 150 other CAR T trials have been registered on ChiCTR. Although CAR T therapy is flourishing in China, there are still some problems that cannot be ignored. In this review, we aim to systematically summarize the clinical practice of CAR T-cell therapy in China. This review will provide an informative reference for colleagues in the field, and a better understanding of the history and current situation will help us more reasonably conduct research and promote cooperation.

## Introduction

With the development of basic research, great advances have been achieved in cancer immunotherapy.^1–4^ Several checkpoint inhibitor drugs and chimeric antigen receptor (CAR) T-cell products have been successfully transformed from bench to bedside.^5–7^ For CAR T cells, the recognition of tumor cells is mediated by CAR molecules so that immune escape with HLA loss in tumor cells can be overcome.^8^ T cells can differentiate into memory T cells, by which the long-lasting antitumor function of CAR T cells can be established.^9,10^ As a T cell-centered cancer immunotherapy, CAR T-cell therapies have exhibited remarkable clinical effects. In 2017, the marketing approval of CTL019 (Novartis) marked the real advent of the CAR T era.

Due to its great potential for continuous optimization, such as better targets, better CAR structure and more efficient preparation process, CAR T-cell therapy has attracted more attention than traditional drugs.

In China, cancer immunotherapy with T cells has been widely used in the clinic. As early as 1988, a modality referred to as lymphokine-activated killer cells^11^ was applied to tumor treatment. Starting in the late 1990s, another T cell-based immunotherapy called cytokine-induced killer (CIK) therapy^12^ gradually became a more popular modality. During this period, other cellular therapies, such as natural killer (NK) cells and dendritic cell-activated CIK (dendritic cells-CIK) cells, have also been widely used. In general, the clinical benefit was minimal, and there was little systematic clinical evidence to support any benefit.

In 2010 and 2011, several important reports suggested the great antitumor function of CAR T cells,^13–15^ which inspired Chinese researchers to carry out domestic CAR T trials. In 2014, the first CAR T clinical trial report from China was released by Han’s team from People’s Liberation Army General Hospital (PLAGH).^16^ In 2015, the Chinese government issued new cellular therapy management policies, which stipulated that cellular therapy could only be carried out in clinical trials.^17^ Henceforth, the nonspecific immunotherapies represented by CIK therapy faded away, and China gradually entered the era of precise immunotherapy represented by CAR T-cell therapy. Since then, CAR T therapy has developed rapidly in China, and in June 2017, China replaced the United States as the country with the most CAR T therapy clinical trials.

Although CAR T therapy has been booming in recent years, many shortcomings still exist. For example, most of the trials are not carried out under an investigational new drug (IND) application, and most clinical trials are small-scaled and single-centered. In addition, many clinical trials are designed without enough rationality and originality.

In this review, we comprehensively summarized the current status of domestic CAR T-cell clinical research to provide references for colleagues in the field. We believe that this is of great significance for promoting cooperation and better understanding the status quo and future trends.

## Overview of the clinical development of CAR T-cell therapy in China

We retrieved trials from the clinicaltrials.gov website using the key words “CAR” or “chimeric antigen receptor”, and manual verification was conducted one by one to exclude non-CAR T-cell therapy trials.

According to our data, the first CAR T clinical trial in the world was launched in the United States in 2003 for the treatment of epithelial ovarian cancer. In China, the first CAR T trial was posted in 2012 by the PLAGH group, which aimed to study the feasibility of CD20-directed CAR T cells in treating patients with lymphoma resistant or refractory to chemotherapy. In other countries, the first CAR T clinical trial was jointly initiated by Germany and the United Kingdom in 2010 to explore the safety and effectiveness of CAR T-19 for childhood acute lymphoblastic leukemia (ALL).

**Fig. 1:**
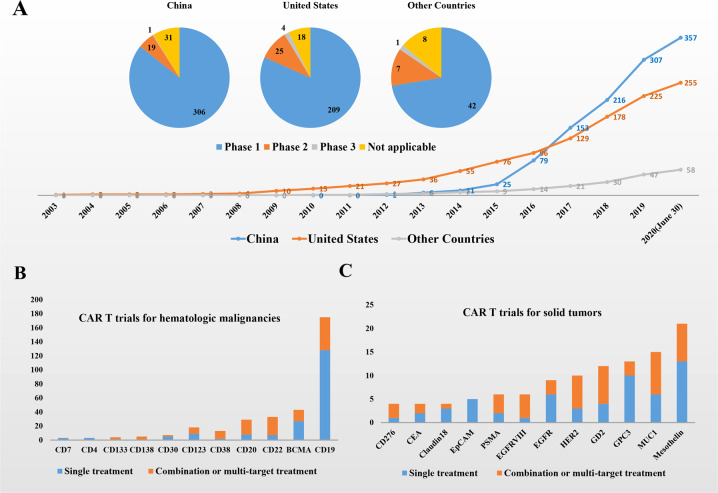
**a** Annual changes in the number of registered CAR T clinical trials and the phases of current trials. **b** Current registered CAR T trials for hematologic malignancies in China. **c** Current registered CAR T trials for solid tumors in China

Since 2013, the number of CAR T clinical trials has begun to grow rapidly around the world. As of January 2015, a total of 57 CAR T clinical trials in the United States had been registered on the clinicaltrials.gov website. In China and other countries, the numbers were 14 and 7, respectively. By January 2016, there were 83 registered CAR T clinical trials in the United States, 27 in China, and 9 in other countries. With rapid development, in June 2017, China (119 registered trials) surpassed the United States (112 registered trials) to become the country with most CAR T therapy clinical trials. To date (June 30, 2020), 357 trials in China, 256 trials in the United States, and 58 trials in other countries have been registered (Fig. 1a, Supplementary Tables 1–3).

Next, we analyzed the data of CAR T clinical trials from China in more detail. As shown in Fig. 1b, CD19 is the most frequently studied target, with 175 trials, including 2 from Taiwan. Among them, 128 clinical trials used CAR T-19 as the only treatment. In addition, CAR T-19 cells were also tested in combination with other treatments, such as CAR T-22 (18 trials) and CAR T-20 (13 trials). B-cell maturation antigen (BCMA) is the second most studied target, with a total of 43 trials. Among them, 27 clinical trials used CAR T-BCMA as the only treatment, and 8 trials intend to evaluate the combination with CAR T-19 therapy. In addition, CD22, CD20, and CD123 are frequently studied targets in CAR T therapy for hematologic malignancies. For solid tumors, mesothelin is the most studied target, with a total of 21 trials (Fig. 1c), of which 8 trials also involved other targets. MUC1 is the next most studied target, involving a total of 14 clinical trials, in which 6 trials used CAR T-MUC1 as the only treatment. In addition, GD2, GPC3, HER2, and epidermal growth factor receptor (EGFR) are also actively studied.

Of the 357 clinical trials in China, 306 trials were phase I studies, 19 trials were phase II studies, and 1 trial entered phase III (registered in 2020); the phases of the remaining clinical trials were not applicable (Fig. 1a). Of the 256 clinical trials in the United States, the numbers of phase I, phase II, and phase III trials were 209, 25, and 4, respectively. Of the 58 clinical trials in other countries, the numbers of phase I, phase II, and phase III trials were 42, 7, and 1, respectively. Most of the clinical trials in China are in early phases, of which the main purpose is to verify safety. This may be because most of the trials in China are investigator-sponsored noncommercial trials, and these projects lack the potential to be developed into drugs.

The Chinese Clinical Trial Registry (ChiCTR) is a national clinical trial registry designated by the Ministry of Health (MOH) to represent China in the World Health Organization International Clinical Trial Registration Platform (WHO ICTRP). The ChiCTR accepts clinical trial registrations implemented in China and around the world; it makes the design of clinical trials and some necessary research information transparent to the public and submits the registered trial information to the WHO ICTRP for global sharing. We also found that many CAR T clinical trials from China have been registered in ChiCTR. In 2015, the first CAR T cell clinical trial was registered. As of August 2020, a total of 139 CAR T cell clinical trials were registered. Of 139 registered clinical trials, 42 trials were phase 0 studies, 59 trials were phase 1 studies, 14 trials entered phase 2, and phases of the remaining clinical trials were not applicable (Supplemental Table 5). In addition, in these registered clinical trials, CD19 is the most frequently studied target, with 81 trials. CD20, CD22, and BCMA are also frequently studied targets in CAR T-cell therapy for hematologic malignancies. For solid tumors, mesothelin, claudin 18.2, GD2, MUC1, and GPC3 are frequently studied targets, and other solid tumor targets, including EGFR, HER2, PSCA, CEA, EGFRvIII, GUCY2C, EpCAM, and TM4SF1, are actively being studied.

**Fig. 2:**
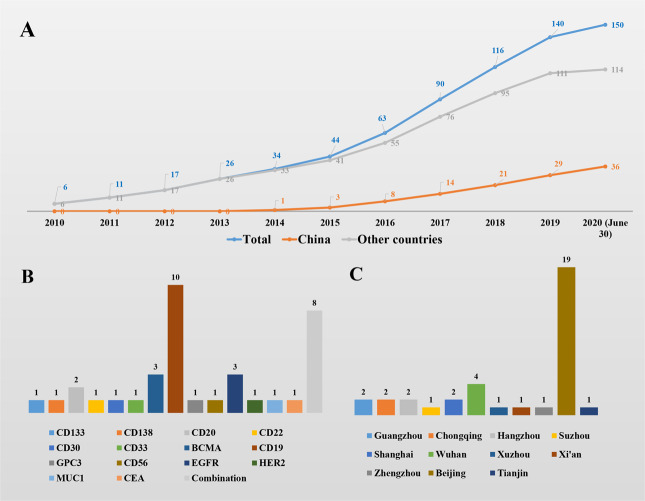
**a** Annual changes in the number of published reports on CAR T therapy. **b** Current published reports of CAR T therapy on different targets in China. **c** Regional distribution of current published reports of CAR T therapy in China

To review the achievements in clinical practice, we searched the PubMed website using the keywords “CAR” and then manually checked the articles. In contrast with most CAR T trials, we found only 36 CAR T clinical reports from China on PubMed as of June 30, 2020 (Table 1). Meanwhile, at least 114 clinical reports from the United States and other countries have been published (Fig. 2a, Supplementary Table 4). This may be due to the late launch of domestic CAR T clinical trials. On the other hand, it also indicates that many domestic CAR T trials lack formal implementation and a long-term layout. According to our data, the first CAR T clinical report in China was published by the group from PLAGH in December 2014.^16^ In this trial, CAR T-20 cells were used to treat patients with B-cell lymphoma. Since 2016, reports of CAR T clinical trials from China have begun to grow steadily. Among the 36 clinical reports, 26 reports are from clinical practice in CAR T therapy for hematological malignancies, and 10 reports are for solid tumors. At present, the team from PLAGH has contributed the most clinical reports, reaching 13.

**Table 1:** The published CAR T clinical reports in China

PMID	Year	Title	Institute	Target
25174587	2015	Treatment of CD33-directed chimeric antigen receptor-modified T cells in one patient with relapsed and refractory acute myeloid leukemia	The General Hospital of the People’s Liberation Army, Beijing	CD33
25444722	2014	Effective response and delayed toxicities of refractory advanced diffuse large B-cell lymphoma treated by CD20-directed chimeric antigen receptor-modified T cells	The General Hospital of the People’s Liberation Army, Beijing	CD20
26451310	2015	Tolerance and efficacy of autologous or donor-derived T cells expressing CD19 chimeric antigen receptors in adult B-ALL with extramedullary leukemia	The General Hospital of the People’s Liberation Army, Beijing	CD19
26961900	2016	Phase 1 clinical trial demonstrated that MUC1 positive metastatic seminal vesicle cancer can be effectively eradicated by modified anti-MUC1 chimeric antigen receptor transduced T cells	Soochow University, Suzhou	MUC1
26968708	2016	Chimeric antigen receptor-modified T cells for the immunotherapy of patients with EGFR-expressing advanced relapsed/refractory non-small cell lung cancer	The General Hospital of the People’s Liberation Army, Beijing	EGFR
27526682	2016	Predominant cerebral cytokine release syndrome in CD19-directed chimeric antigen receptor-modified T-cell therapy	The First Affiliated Hospital, School of Medicine, Zhejiang University, Hangzhou	CD19
27582488	2017	Autologous T cells-expressing CD30 chimeric antigen receptors for relapsed or refractory Hodgkin’s lymphoma: an open-label phase I trial	The General Hospital of the People’s Liberation Army, Beijing	CD30
27887660	2016	Co-infusion of haplo-identical CD19-chimeric antigen receptor T cells and stem cells achieved full donor engraftment in refractory acute lymphoblastic leukemia	The General Hospital of the People’s Liberation Army, Beijing	CD19 with stem cells transplantation
28039267	2017	Potent anti-leukemia activities of chimeric antigen receptor-modified T cells against CD19 in Chinese patients with relapsed/refractory acute lymphocytic leukemia	The First Affiliated Hospital, School of Medicine, Zhejiang University, Hangzhou	CD19
28057014	2017	Cocktail treatment with EGFR-specific and CD133-specific chimeric antigen receptor-modified T cells in a patient with advanced cholangiocarcinoma	The General Hospital of the People’s Liberation Army, Beijing	EGFR and CD133
28366766	2017	Phase I Escalating-Dose Trial of CAR-T therapy targeting CEA(+) metastatic colorectal cancers	Southwest Hospital, Third Military Medical University, Chongqing	CEA
28577043	2017	Anti-CD138 chimeric antigen receptor-modified T-cell therapy for multiple myeloma with extensive extramedullary involvement	Tianjin Medical University Cancer Institute and Hospital, Tianjin	CD138
28710747	2018	Phase I study of chimeric antigen receptor modified T cells in treating HER2-positive advanced biliary tract cancers and pancreatic cancers	The General Hospital of the People’s Liberation Army, Beijing	HER2
29138340	2017	Phase I study of chimeric antigen receptor-modified T cells in patients with EGFR-positive advanced biliary tract cancers	The General Hospital of the People’s Liberation Army, Beijing	EGFR
29263894	2016	Treatment of CD20-directed chimeric antigen receptor-modified T cells in patients with relapsed or refractory B-cell non-Hodgkin lymphoma: an early phase IIa trial report	The General Hospital of the People’s Liberation Army, Beijing	CD20
29458388	2018	A novel generation 1928zT2 CAR T cells induce remission in extramedullary relapse of acute lymphoblastic leukemia	Guangdong General Hospital, Guangzhou	CD19
29503204	2018	In vivo expansion and antitumor activity of coinfused CD28- and 4-1BB-engineered CAR-T cells in patients with B cell leukemia	The Second Affiliated Hospital of Henan University of Traditional Chinese Medicine, Zhengzhou	CD19
29637550	2018	Treatment of acute lymphoblastic leukemia with the second generation of CD19 CAR-T containing either CD28 or 4-1BB	Southwest Hospital, Third Military Medical University, Chongqing	CD19
29900044	2018	CD133-directed CAR T cells for advanced metastasis malignancies: a phase I trial	The General Hospital of the People’s Liberation Army, Beijing	CD133
30348186	2018	Anti-BCMA CAR-T cells for treatment of plasma cell dyscrasia: case report on POEMS syndrome and multiple myeloma	Tongji Hospital of Tongji Medical College, Huazhong University of Science and Technology, Wuhan	BCMA
30572922	2018	A phase 1, open-label study of LCAR-B38M, a chimeric antigen receptor T-cell therapy directed against B-cell maturation antigen, in patients with relapsed or refractory multiple myeloma	The Second Affiliated Hospital of Xi’an Jiaotong University, Xi’an	BCMA
30988175	2019	Exploratory trial of a biepitopic CAR T-targeting B-cell maturation antigen in relapsed/refractory multiple myeloma	Ruijin Hospital affiliated with Shanghai Jiao Tong University School of Medicine, Shanghai	BCMA
31011207	2019	A safe and potent anti-CD19 CAR T-cell therapy	Peking University Cancer Hospital and Institute, Beijing	CD19
31055613	2019	Improving the safety of CAR-T-cell therapy by controlling CRS-related coagulopathy	Union Hospital, Tongji Medical College, Huazhong University of Science and Technology, Wuhan	CD19
31110217	2019	CD22 CAR T-cell therapy in refractory or relapsed B acute lymphoblastic leukemia	Beijing Boren Hospital, Beijing	CD22
31321805	2019	Anti-CD19 chimeric antigen receptor-modified T-cell therapy bridging to allogeneic hematopoietic stem cell transplantation for relapsed/refractory B-cell acute lymphoblastic leukemia: an open-label pragmatic clinical trial	Union Hospital, Tongji Medical College, Huazhong University of Science and Technology, Wuhan	CD19
31378662	2019	A combination of humanized anti-CD19 and anti-BCMA CAR T cells in patients with relapsed or refractory multiple myeloma: a single-arm, phase 2 trial	the Affiliated Hospital of Xuzhou Medical University, Xuzhou	CD19 and BCMA
31489688	2019	Shortening the ex vivo culture of CD19-specific CAR T-cells retains potent efficacy against acute lymphoblastic leukemia without CAR T-cell-related encephalopathy syndrome or severe cytokine release syndrome	Zhujiang Hospital, Southern Medical University, Guangzhou	CD19
31651858	2019	CD56-chimeric antigen receptor T-cell therapy for refractory/recurrent rhabdomyosarcoma: a 3.5-year follow-up case report	Beijing Children’s Hospital, Capital Medical University, Beijing	CD56
31697824	2020	Efficacy and safety of CAR19/22 T-cell cocktail therapy in patients with refractory/relapsed B-cell malignancies	Tongji Hospital, Tongji Medical College, Huazhong University of Science and Technology, Wuhan	CD19 and CD22
31725148	2020	Sequential CD19-22 CAR T therapy induces sustained remission in children with r/r B-ALL	Beijing Boren Hospital, Beijing	CD19 and CD22
32321169	2020	Early response observed in pediatric patients with relapsed/refractory Burkitt lymphoma treated with chimeric antigen receptor T cells	Beijing Boren Hospital, Beijing	CD19 and CD20 or CD22
32371538	2020	Chimeric Antigen Receptor-Glypican-3 T-cell therapy for advanced hepatocellular carcinoma: results of Phase I Trials	Renji Hospital, Shanghai Jiao Tong University School of Medicine, Shanghai	GPC3
32527643	2020	Anti-EGFR chimeric antigen receptor-modified T cells in metastatic pancreatic carcinoma: a phase I clinical trial	The General Hospital of the People’s Liberation Army, Beijing	EGFR
32536980	2020	Co-infusion of high-dose haploidentical donor cells and CD19-targeted CART cells achieves complete remission, successful donor engraftment and significant CART amplification in advanced ALL	The General Hospital of the People’s Liberation Army, Beijing	CD19 with stem cells transplantation
32556247	2020	Optimized tandem CD19/CD20 CAR-engineered T cells in refractory/relapsed B-cell lymphoma	The General Hospital of the People’s Liberation Army, Beijing	CD19 and CD20

## Hematological malignancies

### CD19

CD19 is expressed almost throughout the development of B cells, and the side effects caused by B cell clearance are clinically controllable. Therefore, CD19 has become an ideal target for CAR T-cell therapy to treat B-cell malignancies. CART-19 is currently the most recognized and widely used CAR T product, and most improvements in CAR T treatment are achieved in the clinical practice of CAR T-19.

In 2010, the City of Hope Medical Center first reported a case of CART-19 treatment. CAR T cells rapidly decayed in vivo due to the overlong in vitro cultivation (up to 55 days), which indicated the necessity of shortening the culture time.^18^ The CAR T-19 clinical practice from Rosenberg’s group (in 2010) and Brentjens’ group (in 2011) initially suggested that chemotherapy pretreatment was vital to fully exert the antitumor function of CAR T cells.^13,14^ In 2011, Carl H. June’s team reported a case of CAR T-19 treatment for refractory chronic lymphocytic leukemia with a low dose (1.5 × 10^5^/kg).^15^ After infusion, the CAR T cells expanded effectively, leading to sustained complete remission (CR), which suggested that in vivo proliferation rather than an infusion dose of CAR T cells was related to clinical response. In addition, the establishment of effective recognition and the management of cytokine release syndrome (CRS) and immune effector cell-associated neurotoxicity syndrome (ICANS) caused by CAR T treatment, as well as the understanding of the recurrence and resistance mechanisms, were also mainly derived from the clinical practice of CAR T-19.^2,19–23^

In China, there are 173 registered CAR T trials involving CD19 so far. The first clinical trial targeting CD19 in China was initiated by the PLAGH team in May 2013 for relapsed and/or chemotherapy refractory B-cell malignancies. Of these trials, 128 used CAR T-19 as the only treatment to specifically study the clinical responses to CAR T-19 therapy.

In April 2016, the Southwest Hospital posted a clinical trial of sequential therapy with CAR T-19 and CAR T-20 cells for diffuse large B-cell lymphoma (DLBCL) to explore the feasibility of multitarget CAR T therapy in preventing recurrence and improving clinical efficacy. Since then, a number of multitarget CAR T therapies based on CAR T-19 have been actively explored in China, including 18 trials with CAR T-22 and 13 trials with CAR T-20. There are different strategies to achieve the combination of different targets, such as the sequential infusion of different CAR T cells, the administration of pooled different CAR T cells and treatment with dual-targeted CAR T cells.^24^

In addition, eight posted trials intended to study the feasibility of CAR T and hematopoietic stem cell transplantation (HSCT) combined therapy. Except for one clinical trial that is planning to study the feasibility and safety of concomitant therapy with CAR-T cells and HSCT in patients with R/R leukemia, the other trials only used CAR T-19 treatment to bridge HSCT.

For clinical efficacy, a total of 16 CAR T-19 clinical reports from China have been published. The first domestic CAR T-19 clinical data were reported by Dai et al.^25^ In this phase I clinical trial, nine patients with relapsed or chemotherapy-refractory B-ALL were treated, and six patients had extramedullary involvement. Objective clinical responses, including those in both the hematopoietic system and extramedullary tissues, were observed in six patients. For the first time, this report demonstrated that CAR T-19 therapy was effective against extramedullary B-ALL, but that donor-derived CAR T cells could cause graft-versus-host disease.

In 2016, Huang’s team reported a case and confirmed that CAR T cells can penetrate the blood–brain barrier to trigger cerebral CRS.^26^ In this case, 1 patient with R/R ALL achieved CR with minimal residual disease (MRD) negative after CAR T-19 treatment. In addition, in 2016, Cai et al.^27^ treated a patient with relapsed and refractory (R/R) ALL with a co-infusion of haplo-identical donor-derived CAR T-19 cells and mobilized peripheral blood stem cells. In this case, CR was achieved, and full donor cell engraftment was also established. This successful case preliminarily suggested that the co-infusion of CAR T cells and hematopoietic stem cells could achieve tumor clearance and hematopoietic reconstruction simultaneously. In a subsequent study, two more patients with advanced B-ALL were treated with the same strategy, and both achieved CR and full donor chimerism.^28^

In 2017, Hu et al.^29^ released their phase I clinical results of the use of CAR T-19 cells to treat R/R ALL. They enrolled 15 patients, of which 12 patients achieved CR 1 month after CAR T-19 cell infusion. However, relapse after CR remained the main obstacle.

In 2018, Weng et al. reported that the antitumor function of CAR T cells could be boosted by the incorporation of the Toll/interleukin-1 receptor (ITR) domain of Toll-like receptor 2 (TLR2). This novel CAR T cell, termed 1928zT2 CAR T, completely eradicated leukemia in three R/R ALL patients.^30^ In another trial, Li et al.^31^ treated ten R/R ALL patients with second-generation CAR T-19 cells modified with the CD28 or 4-1BB costimulatory motif. In the CD28 group, three out of five patients achieved CR, and one patient achieved partial remission (PR). In the 4-1BB group, three out of five patients achieved CR. However, five out of the six CR patients relapsed within 2–8 months after infusion, and relapsed leukemia was verified to be CD19 positive.

In 2019, Ying et al.^32^ modified native CAR molecules with the addition of 15 amino acids to the hinge and transmembrane domains, leading to reduced cytokine production and T-cell proliferation without decreasing antitumor function. This novel CAR structure was then tested in 25 patients with advanced B-cell non-Hodgkin lymphoma (B-NHL). Among the 25 patients, 7 patients achieved CR, and 8 patients achieved PR. No significant CRS or ICANS was observed in any of the treated patients. In another clinical report, Jiang et al.^33^ enrolled 53 R/R B-ALL patients who received split infusions of CAR T-19 cells. Forty-seven out of the 53 patients successfully achieved CR, and 6 patients showed no response to CAR T therapy. Twenty-one patients who had achieved CR suffered relapse between 1.1 and 15.6 months after CAR-T therapy. According to the follow-up reports of the same clinical trial, a total of 51 out of 58 patients achieved CR in this trial, and 21 MRD-negative CR patients received consolidative allo-HSCT within 3 months after CAR-T therapy.^34^ Event-free survival and relapse-free survival were significantly prolonged by allo-HSCT, but no difference in overall survival was observed. In addition, this study indicated that shortening the cultivation (7 days) is feasible and promising. In another trial posted by Tu et al. in 2019, a total of 25 adult patients with R/R ALL were infused with CAR T-19 cells. Twenty-two patients achieved CR, and 1 patient achieved PR.^35^ As a mature modality, CAR T-19 therapy has been increasingly tested in combination with other targets. In 2019, Xu’s team assessed the safety and activity of a combination of CAR T-19 and CAR T-BCMA cells in patients with R/R multiple myeloma (MM).^36^ According to the report, 12 out of 21 treated patients achieved CR, and 8 patients achieved PR. This demonstrated that dual CAR T cell combinations might be a promising strategy.

In 2020, a pilot study enrolling 89 patients with R/R B-cell malignancies to evaluate the efficacy and safety of the sequential infusion of CAR T-19 and CAR T-22 cells was posted.^37^ Forty-nine out of the 51 patients with ALL achieved MRD-negative CR. Eighteen out of the 38 patients with B-NHL achieved CR, and 8 patients achieved PR. CD19-negative relapse was found in only 1 patient, which indicated that the sequential infusion of CAR T cells may have reduced the rate of antigen-escape relapse. In addition, in 2020, another strategy for target combination was also proven to be effective. In Pan’s report, 20 children with R/R ALL were enrolled, and all of them achieved MRD-negative CR after CAR T-19 treatment.^38^ Following CAR T-22 treatments, instead of HSCT, the patients were in MRD-negative remission, leading to 17 patients achieving at least 1-year leukemia-free survival (LFS), which represented a significant improvement in long-term remission over that seen with single CAR T treatments. These data demonstrated that the safety and feasibility of sequential infusion may lead to an improved LFS without the necessity of consolidative HSCT. In another trial reported in 2020, CAR T-20 or CAR T-22 cells were given to R/R B-cell lymphoma patients who did not achieve CR after initial CAR T-19 treatment, and three of them achieved CR.^39,40^ For the first course of CAR T-19 treatment, the overall CR rate (CRR) was 41.7% (7/17). Expressing dual-targeted CAR molecules (tandem CARs) is another combination strategy. In 2020, Tong et al.^41^ found that optimized tandem CAR T-19/20 cells could form a more stable immunological synapse, leading to better antitumor function. The dual-targeted CAR T cells were then tested in R/R NHL patients, and a total of 28 patients received CAR T-19/20 treatment. Using this dual-targeted CAR T therapy, up to 71% of patients achieved CR, and two patients achieved PR. In addition, the progression-free survival (PFS) rate at 12 months was as high as 64%. In summary, the safety and effectiveness of CAR T-19 therapy in the treatment of ALL, B-NHL, and MM have been gradually confirmed by Chinese researchers. Meanwhile, different strategies to enhance the clinical benefits and different application scenarios of CAR T-19 therapy are actively being explored. There may be more patients who have been treated with CAR T-19 therapy in reality, and these data from multiple institutions could be very meaningful to enrich our understanding.

### CD20

The expression pattern of CD20 is very similar to that of CD19, except that CD20 is absent in pro-B cells. Being present in 90% of B-cell lymphomas, it is a well-studied target for B-NHL treatment, and anti-CD20 antibody drugs have shown significant clinical effects for B-NHL. Its long-term application and continuous upgrade in clinical products may be largely due to the favorable features of CD20.

The first CAR T-20 clinical trial in China was carried out by the PLAGH team in November 2012 and was also the first formal CAR T trial in China. In this trial, CAR T-20 cells were tested for the treatment of lymphoma that was resistant or refractory to chemotherapy. It was not until March 2016 that the second Chinese CAR T-20 clinical trial was registered by Southwest Hospital. At present, a total of 29 CAR T trials involving CD20 have been registered. However, only eight trials used CAR T-20 cells as the single treatment for B-cell lymphoma, and CAR T-20 cells tended to be used in combination with other CAR T cells to improve the clinical efficacy.

A total of three clinical reports on CAR T-20 therapy were found. In an article published in 2020, CAR T-20 was used as the second course of treatment for patients who relapsed after initial CAR T-19 treatment.^39,40^ The other two reports are from the same clinical trial conducted by the PLAGH group (NCT01735604). In an earlier study, two patients without bulky tumors achieved CR and a 6-month remission. Five patients with bulky tumors were treated with CAR T-20 and conditioning chemotherapies, and four of them were evaluable for clinical efficacy.^16^ In the follow-up study of this clinical trial, another 11 patients were enrolled, and the overall objective response rate was 81.8% (9 of 11), with 6 patients achieving CR and 3 patients achieving PR.^42^ These results demonstrated the feasibility of CAR T-20 therapy for B-cell lymphomas.

### CD22

CD22 is predominantly expressed on the surface of mature B cells and most B-cell-derived tumors, which makes it another hot target for the treatment of autoimmune diseases and B-cell malignancies. At present, many immunotherapeutic drugs targeting CD22, such as monoclonal antibody drugs, antibody-drug conjugates (ADCs) and CAR T-22 cells, are being developed, and CD22-ADC has been approved for clinical use.

Xinqiao Hospital registered the first domestic CAR T-22 clinical trial in March 2016. In this trial, CAR T-20 therapy was used to treat patients with lymphoma that was refractory or resistant to CAR T-19 treatment. At present, a total of 33 Chinese CAR T trials involving CD22 could be found on the ClinicalTrials.gov website. Similar to CAR T-20 cells, CAR T-22 cells are more frequently used in combination with other treatments (26/33), and only 7 trials have been dedicated to the study of CAR T-22 cell safety and ability.

By searching PubMed, we found four clinical reports related to CAR T-22. In three of these reports, as a combined modality, the main aim of CAR T-22 therapy was to improve the clinical efficacy of traditional single-target CAR T-19 therapy by reducing the recurrence rate or salvaging patients relapsed from CAR T-19 therapy.^37–39^ In an article published in 2019, as a single treatment, the antitumor function of CAR T-22 was strictly evaluated.^43^ This trial was conducted by a group from Beijing Boren Hospital, and 34 R/R ALL pediatric and adult patients who failed CAR T-19 therapy were enrolled. Among 30 evaluable patients, 24 patients (80%) achieved CR. Although CD22 loss or mutation was not observed in relapsed patients, CD22 has been proven to be more prone to silencing by signaling pathways or epigenetic modification.^44^ Given that signaling or epigenetic regulation generally occurs faster and more frequently than mutations, we believe that CD22 may not be an ideal target compared with CD19.

### BCMA

BCMA is a member of the tumor necrosis factor superfamily. It is widely expressed on MM cells, with little expression detected on normal cells.^45^ Therefore, BCMA is viewed as an ideal target for MM treatment. Compared with other commonly used targets, CAR T clinical trials targeting BCMA have been carried out later. According to our data, the first CAR T clinical trial involving BCMA in the world was posted in September 2015. It was not until November 2016 that Chinese researchers launched the first domestic CAR T-BCMA clinical trial. Before 2018, there were only 7 clinical trials involving BCMA in China. However, BCMA has become the most actively studied target in recent years due to the impressive antitumor efficacy against MM presented by several studies. To date, 43 CAR T clinical studies involving BCMA have been carried out in China. Notably, 27 of these trials used CAR T-BCMA therapy as the only treatment to strictly evaluate its antitumor activity or safety. In addition, the only CAR T clinical study in China that has entered phase III is a CAR T trial targeting BCMA (NCT04287660).

In the clinical research of CAR T-BCMA, China has been at the forefront of the world. There are currently eight CAR T-BCMA clinical reports in the world, four of which are from China. In 2018, Xu et al.^46^ reported their clinical practice of CAR T-BCMA therapy for POEMS (polyneuropathy, organomegaly, endocrinopathy, monoclonal gammopathy, and skin changes) syndrome and R/R MM. One patient with incapacitating POEMS syndrome and one patient with R/R MM received CAR T-BCMA cells, and both achieved stringent CR. For the first time, CAR T-BCMA was proven to be a feasible therapeutic option for patients with POEMS syndrome.

In a phase I multicenter study sponsored by Nanjing Legend Biotech Inc., the safety and efficacy of LCAR-B38M, a dual epitope-binding CAR T-BCMA cell product, in patients with MM were evaluated systematically. According to the earlier released result, 57 enrolled patients received LCAR-B38M treatment.^47^ The overall response rate (ORR) was 88%, and 39 patients (68%) achieved CR, 3 patients (5%) achieved very good PR and 8 patients (14%) achieved PR. In the later released results, 17 patients with R/R MM were enrolled and treated.^48^ The ORR was 88.2%, with 13 patients achieving stringent CR, 2 patients reaching very good PR, and 1 patient not responding to the therapy. Overall, these results demonstrated that CAR T-BCMA was a promising therapy for R/R MM with manageable adverse events in most cases.

In addition, the activity and safety of CAR T therapy targeting BCMA combined with CAR T-19 cells in patients with R/R MM were also assessed, which has been discussed above.^36^

### Other targets

In addition to CD19, CD20, CD22, and BCMA, other targets, such as CD30, CD123, CD33, CD38, and CD138, have been explored in CAR T therapies for different hematological tumors. For these targets, most of the relevant clinical trials are exploratory, and Chinese researchers have done much pioneering work.

CD33, also referred to as Siglec-3, is a transmembrane receptor expressed on cells of myeloid lineage that is expressed in more than 90% of patients with acute myeloid leukemia (AML).^49^ Therefore, it may be a potential target for the treatment of AML. Currently, ten CAR T clinical trials involving CD33 have been registered in China. In 2013, the PLAGH group registered the world’s first CAR T clinical trial targeting CD33 to study the feasibility of CAR T-33 therapy for R/R AML. In 2014, a case report from this trial was published, which is also the first CAR T clinical study report in China.^50^ A marked decrease in blasts in bone marrow was observed within 2 weeks after therapy, accompanied by significant but manageable adverse events.

CD30, also known as TNFRSF8, is a cell membrane protein of the tumor necrosis factor receptor family that is widely expressed in Hodgkin’s lymphoma.^51^ Anti-CD30 antibody drugs have been successfully developed for the treatment of Hodgkin’s lymphoma.^52^ Currently, seven CAR T trials involving CD30 have been registered in China. The first domestic CAR T-30 clinical trial was carried out by the PLAGH group in October 2014, and the results of this trial were released in 2017. In this trial, 18 patients with R/R Hodgkin lymphoma were enrolled and treated, most of whom had heavy treatment histories or multiple tumor lesions.^53^ CAR T-30 treatments were well tolerated with grade ≥3 toxicities only in two patients. For clinical response, seven patients achieved PR, and six patients achieved stable disease (SD). Notably, lymph nodes presented a better response than extranodal lesions. These results warranted further large-scale patient recruitment.

CD138, also known as syndecan-1, is a transmembrane proteoglycan mainly confined to the late stages of B-cell differentiation. CD138 is also highly expressed on MM cells, making it an attractive therapeutic target for MM treatment.^54^ In June 2013, the first Chinese CAR T-138 clinical trial was registered. To date, there are five registered clinical trials in China involving CD138. In a CAR T-138 trial conducted by the PLAGH group, five patients with chemotherapy-refractory MM were enrolled and treated.^55^ The treatments were well tolerated; four patients had SD longer than three months, and a significant reduction of myeloma cells in peripheral blood was observed in one patient. In another trial conducted by Tianjin Medical University Cancer Institute and Hospital, a patient with refractory MM received CAR T-138 treatment. With extramedullary involvement, this patient achieved PR.^56^

In addition, some original preclinical studies on novel targets, such as CD38 (with 13 registered CAR T clinical trials),^57^ CD123 (with 18 registered CAR T clinical trials),^58,59^ CD4 (with 3 registered CAR T clinical trials),^60^ and CD7 (with 3 registered CAR T clinical trials)^61^ have also been reported. We believe that in the future, more CAR T clinical studies for different hematological tumors will be reported.

## Solid tumors

The earliest CAR T clinical trial and clinical report are both about solid tumors.^62,63^ However, the progress of CAR T therapy in solid tumors has not gone well. For solid tumors, the heterogeneity and immunosuppressive microenvironment are viewed as the main factors limiting the benefits of CAR T cell treatment for solid tumors.^64^ In addition, physical barriers could further reduce the infiltration of CAR-T cells in the tumor bed.^65^ These issues have been extensively discussed.

To date, 105 Chinese CAR T clinical trials for solid tumors have been registered on the clinicaltrials.gov website, and 10 domestic published clinical studies could be found on PubMed. In addition, many preclinical studies reported by Chinese researchers have suggested some potential strategies to improve the safety and efficiency of CAR T therapy for solid tumors.

### HER2

HER2, an oncogene encoding a growth factor receptor, is a transmembrane glycoprotein that regulates cell proliferation and differentiation.^66,67^ Numerous studies have indicated the overexpression of HER2 in many epithelium-derived malignancies.^63,68–71^ Therefore, HER2 is viewed as a promising target for many solid tumors.^72^ To test the antitumor potential of CAR T therapy for HER2-positive tumor cells, ten CAR T clinical trials involving HER2 were carried out in China, among which the first one was posted in September 2013. However, the strict evaluation of CAR T-HER2 was only implemented in three trials, in which CAR T-HER2 was the only treatment.

In a phase I clinical trial, CAR T-HER2 single therapy was tested for advanced biliary tract cancer (BTC) and pancreatic cancer (PC). Eleven patients were enrolled and treated with CAR T-HER2 cells, among which one achieved PR for 4.5 months and five achieved SD.^73^ Compared with a clinical trial performed by the Baylor College of Medicine,^74^ the clinical responses were similar, and these results indicated the safety and feasibility of anti-HER2 CAR T cells in treating solid tumors.

### Epidermal growth factor receptor

EGFR, also known as human epidermal receptor 1 (HER1), is a receptor tyrosine kinase expressed in both epithelial cells and epithelium-derived malignancies.^75–77^ In addition, the gene amplification and mutation of EGFR can result in abnormal cell proliferation and survival, metastasis, and tumor-induced neoangiogenesis.^78^ To test the feasibility of EGFR-targeted CAR T therapy for different epithelium-derived tumors, many clinical trials have been conducted. In China, 9 CAR T clinical trials have been posted, of which 6 trials intend to use CAR T-EGFR as the single treatment to evaluate its safety and ability.

The first registered CAR T-EGFR trial was posted in June 2013, and the results were released in 2016. In this trial, patients with EGFR-positive (>50%) R/R non-small lung cancer (NSCLC) received CAR T-EGFR cell infusion. Of the 11 evaluable patients, 2 patients achieved PR, and 5 had SD.^79^ In the same trial, CAR T-EGFR treatment was further explored for the treatment of advanced BTC and PC.^80–82^ For BTC, 19 patients (14 cholangiocarcinomas (CCA) and 5 gallbladder carcinomas) with EGFR-positive (>50%) advanced unresectable, relapsed/metastatic BTC were treated with CAR T-EGFR cells. The treatments were well tolerated, with only three patients suffering grade ≥3 acute fever/chills. Of 17 evaluable patients, 1 patient achieved CR, and 10 patients achieved SD. In another case, a 52-year-old female with advanced unresectable/metastatic CCA received successive infusion of CAR T-EGFR and CAR T-133 (CD133 targeted). CD133 is expressed in various carcinomas, including CCA, and serves as a specific molecular biomarker for cancer stem cells (CSCs). Therefore, subsequent CAR T-133 therapy may benefit patients by killing CCA cells and clearing CSCs. Finally, the patient achieved an 8.5-month PR from CAR T-EGFR therapy and a 4.5-month PR from CAR T-133 treatment. However, CAR T-133 cells induced rapidly deteriorating grade 3 systemic subcutaneous hemorrhages and congestive rashes together with CRS, which needed emergent intervention, including intravenous methylprednisolone. This may be due to the widespread expression of CD133 in different cells, especially stem cells. For PC, 16 patients were enrolled and received CAR T-EGFR infusion after the conditioning regimen. Grade ≥3 adverse events mainly included fever/fatigue, nausea/vomiting, mucosal/cutaneous toxicities, pleural effusion, and pulmonary interstitial exudation, and these events were reversible. Of 14 evaluable patients, 4 achieved PR, and 8 had SD for 2–4 months.

### GPC3

GPC3, glypican-3, is a heparan sulfate proteoglycan that is highly expressed in a variety of solid tumors, especially in hepatocellular carcinoma (HCC), while its expression is very low in normal adult tissues. GPC3 has been indicated as a prognostic marker for HCC, suggesting that GPC3 is a rational immunotherapeutic target for HCC.^83^ In March 2015, a group from Renji Hospital registered China’s first and the world’s first phase I clinical trial using CAR T-GPC3 to treat HCC. To date, 13 clinical trials involving CAR T-GPC3 have been registered in China.

Recently, the results of a clinical trial conducted by Renji Hospital were released. A total of 13 adult patients with advanced GPC3-positive HCC received CAR T-GPC3 therapy following lymphodepletion.^84^ Eight out of the 13 patients experienced reversible CRS (grade 1/2), and 1 patient experienced severe CRS (grade 5). Two PR responses were confirmed, and a sustained SD response (ongoing after 44.2 months) was achieved in 1 patient. The safety of CAR T-GPC3 therapy was demonstrated, and early signs of the antitumor activity of CAR T-GPC3 cells against advanced HCC were also observed.

### CD133

CD133, a pentaspan transmembrane glycoprotein, is overexpressed in various solid tumors, including HCC, PC, gastric cancer, and intrahepatic cholangiocarcinomas.^85,86^ Moreover, the high expression of CD133 in HCC cells corresponds with higher stage tumors, indicating a poor prognosis for most patients.^87,88^ These results indicate that CD133 is a reasonable target for immunotherapy in patients with advanced CD133-positive tumors. The first CAR T-33 clinical trial was posted in September 2015 by PLAGH. A total of four trials involving CD133 could be found on the clinicaltrials.gov website, and all of them were from China. In 2018, the results of the CAR T-133 trial conducted by PLAGH were released.^89^ They enrolled 23 patients (14 with HCC, 7 with PC, and 2 with colorectal carcinomas). The eight initially enrolled patients with HCC were treated by a CART-133 cell dose escalation scheme (0.05–2 × 10^6^/kg), and an acceptable cell dose (0.5–2 × 10^6^/kg) was determined for the later enrolled patients. The toxicity was generally self-recovered. Of the 23 patients, 3 achieved PR, and 14 achieved SD. The antitumor activity of CAR T-133 cells was clearly confirmed by the immunohistochemistry of biopsy tissues, showing that CD133-positive cells were eliminated after CAR T-133 infusions.

### MUC1

MUC1, or Mucin 1, is expressed on the apical surface of some epithelial cells to prevent pathogens from reaching the cell surface. MUC1 also functions in cell signaling, and the aberrant overexpression of MUC1 has been detected in many solid tumors.^90^ Therefore, MUC1 is generally viewed as a tumor-associated antigen that might be a potential target for CAR T therapy in some solid tumors. MUC1-targeted CAR T therapies have been involved in 15 clinical trials in China, of which the first trial was registered in October 2015 by PersonGen BioTherapeutics.

In 2016, a case from this trial was reported, which was the first CAR T-MUC1 clinical report in the world.^91^ In this study, a patient with MUC1-expressing seminal vesicle cancer was treated with two kinds of CAR T cells, including CAR T cells modified with IL-12 expression to overcome the immune suppressive microenvironment and CAR T cells with mutated scFv to improve binding affinity. These two kinds of CAR T cells were injected intratumorally into two separate lesions. A new necrotic area that perfectly matched the injection site of CAR T-MUC1 was detected. The adverse events of this treatment were generally mild and reversible.

### CEA

CEA, or carcinoembryonic antigen, has been regarded as a sensitive tumor biomarker for gastrointestinal cancers. Importantly, CEA is not expressed in most normal adult tissues, except in the gastrointestinal tract at a low level.^92^ Therefore, CEA can be a potent target for CAR T-cell therapy for gastrointestinal cancers.

In 2017, the first CAR T-CEA clinical report was released by Southwest Hospital.^93^ In this study, 10 patients with CEA-positive R/R colorectal cancer were enrolled. Five dose levels of CAR T cells were tested, and all levels were well tolerated. Seven patients achieved SD, and 2 patients remained with SD for more than 30 weeks. The tumor shrinkage evaluated by imaging and the decrease in serum CEA clearly indicated the antitumor function of CAR T-CEA cells. These results were from a phase I clinical trial registered in January 2015, which was also the first posted CAR T-CEA trial in the world. To date, four trials involving CEA have been conducted in China.

### CD56

CD56, a member of the immunoglobulin superfamily, is a biomarker of nervous system cells and NK cells. In skeletal muscle tumors and peripheral neurogenic tumors, CD56 is significantly overexpressed.^94^ In a case reported in 2019, a 2-year-old patient with rhabdomyosarcoma (RMS) received CAR T-56 treatment. The treatment led to 3.5 years of ongoing CR.^95^ Overall, this case indicated that CAR T-56 cell therapy was a safe and effective approach for the treatment of R/R RMS.

To date, five registered trials involve CD56, but all these trials are for AML or MM.

### Mesothelin

Mesothelin (MSLN), a cell–surface glycoprotein, is a tumor-differentiation antigen that is highly expressed in mesothelioma (MPM), lung cancer, PC, and ovarian cancer.^96–99^ However, it is also expressed normally in the pleura, pericardium, and peritoneum mesothelial cells.^100^

In 2014, a clinical study on anti-MSLN CAR T cells modified with mRNA for the short-term expression of CAR was reported.^101^ In this report, the antitumor effect and safety of CAR T-MSLN were suggested in 1 patient with malignant pleural MPM and 1 patient with malignant PC. The mRNA-modified CAR T cells were further tested in six patients with metastatic PC, and PFS times of 3.8 and 5.4 months were observed in two patients. The safety of CAR T-MSLN therapy was guaranteed by transient CAR expression.

To date, no clinical data on CAR T-MSLN have been published in China. However, 21 CAR T clinical trials involving MSLN have been registered by Chinese researchers, which makes MSLN the most studied target for solid tumor treatment.

### Other targets for solid tumors

In addition to the targets mentioned above, many other targets for solid tumors are being tested in domestic clinical trials. GD2 is also a frequently studied target, and currently, 12 domestic clinical trials involving GD2 have been posted. The other studied targets include EphA2, EpCAM, EGFRVIII, HER4, IGFR1, Lewis-Y, LMP1, CD70, Claudin18.2, PSMA, FTα, PSCA, MAGE, ROR2, AXL, IL13R, NY-ESO-1, DR5, C-met, FAP, and Nectin4.

## The regulation and commercialization of CAR T therapy in China

Due to the uniqueness of living cell therapies, the policies on CAR T-cell therapy in China have been revised many times. Since 2009, all autologous cell therapies, including CAR T-cell therapy, were allowed to be carried out in certain hospitals with the permission of the MOH. This also means that CAR T therapy was regulated as a medical technology during that period. However, no hospital had ever received official permission from the MOH, and the chaotic application of cell therapy has brought much criticism to this field and seriously disrupted the industry. In 2015, the MOH halted the unregulated application of cell therapy in the clinic, and cell therapies could only be carried out in the form of clinical trials. For projects that are planned to be developed into drugs, the relevant IND application must be submitted to the China Food and Drug Administration (CFDA). On December 11, 2017, the China FDA announced the acceptance of the first IND application of CAR T therapy, which was from Nanjing Legend Biotechnology Co., Ltd. As of July 30, 2020, according to the information on the CDE website, 33 CAR T clinical trials have been accepted by the CDE, involving 25 products from 17 companies. Among the 33 clinical studies, CD19 is the most studied target, with 23 trials, and BCMA is the second most popular target, with 6 trials. In addition, 4 clinical trials selected CD30, claudin 18.2, GPC3 and gp120 as the studied targets. Among them, the LCAR-B38M product from Nanjing Legend has been included in the breakthrough therapy category, which is also the only one at present. According to the new regulations of CDE, within 60 days from the date of acceptance and payment of the application, if the applicant does not receive a negative or challenged opinion from the CDE, the clinical trial can be carried out in accordance with the submitted plan.

CAR T-cell therapy is a complex process, including patient recruitment and enrollment and cell manufacture, delivery and infusion, and it is a significant challenge for institutions to deliver this therapy as a standard of treatment to eligible patients. Simply using the resources of hospitals is not enough to treat more tumor patients, and it is necessary that companies join to solve the problem. Therefore, the active participation of companies is essential for the commercialization of CAR T therapy.

China, Europe, and America all attach great importance to technology research on CAR T cells. Two commercially available CAR T cells, tisagenlecleucel produced from Novartis and axicabtagene ciloleucel produced from Kite Pharma, have been recently approved by the U.S. Food and Drug Administration (listed on the www.fda.gov website). In China, a series of technology start-ups, such as Nanjing Legend Biotechnology Co., Ltd., Cellular Biomedicine Group, and Fosun Kite Biotechnology Co., Ltd. have been founded in recent years to provide qualified CAR T cells for the treatment of tumor patients. For these commercial CAR T cell companies, it is of utmost importance to establish a rigorous and specific infrastructure that meets clinical, administrative, and regulatory demands.

The logistics of CAR T cell delivery are complex, as cell products require timely long-distance transfer between hospitals and industries. Therefore, the delivery of commercial CAR T cells into clinical cancer treatment requires detailed and careful planning, resource allocation, and the utilization of existing infrastructure. Several issues should be addressed before and after commercial CAR T cell delivery, including the following: (1) CAR T cell research and development in vitro and in animal models by hospitals or companies or both; (2) hospital ethics approval by the committee and registration on www.chictr.org.cn or www.clinicaltrials.gov; (3) patient recruitment and enrollment; (4) CAR T cell manufacture in a Good Manufacturing Practice facility according to the standard operating procedures supervised by hospitals and companies; (5) bridge between hospitals and companies on peripheral blood collection and CAR T cell delivery; (6) management of CAR T cell infusion and post-infusion care; and (7) financial and clinical data implications.

## Discussion

CAR T therapy has achieved remarkable success in the treatment of hematological tumors, and many therapies are being actively transformed into products. In the future, targets that can be used to treat myeloid leukemia effectively still need to be explored. For ALL, MM, and B-cell lymphoma, CAR T therapy targeting CD19 and BCMA has achieved high CR rates. In the future, more efforts should be put into strategies that can reduce relapse after CR, which can be achieved through multitarget combination or combination with other treatment methods (such as HSCT). In addition, how to manage the adverse effects caused by CAR T therapy is also an important issue that needs more exploratory research in the future.

Although CAR T therapy is facing more problems in the treatment of solid tumors, its antitumor function is beyond doubt.^102,103^ In our opinion, several strategies that can improve the effectiveness of CAR T therapy for solid tumors are worth exploring. For target selection, higher tumor coverage could be achieved by using multitarget CAR T therapy, which can ensure that more tumor cells can be eliminated. However, higher coverage may also lead to more off-target cytotoxicity. Therefore, more careful evaluation is needed before clinical implementation. To avoid off-target cytotoxicity, split CAR^104^ and inhibitory CAR^105^ can be used, but this will unavoidably reduce the coverage of tumor cells. This dilemma reminds us that in the practice of CAR T cell treatment of solid tumors, inducing endogenous tumor-specific immune responses through CAR T-cell therapy should be a more potential strategy. This concept has been confirmed in many preclinical studies and clinical cases.^106–108^

In addition, improving the antitumor function of CAR T cells is still a core issue. It mainly includes improving in vivo proliferation, reducing exhaustion, and enhancing infiltration ability and resistance to the immunosuppressive microenvironment. For example, the expression of IL-12 can endow CAR T cells with better in vivo proliferation and persistence;^109,110^ the overexpression of CXCR2 can improve the chemotaxis of T cells to tumor tissues;^111^ and the construction of PD1-CD28 chimeric receptors can transform immunosuppressive signals into activated T cell signals.^105^ With the specificity and flexibility of antibodies combined with the long-term survival and efficient killing ability of T cells, CAR T cells hold great potential for diseases other than tumors. Among them, the concept of CAR T therapy for the treatment of human immunodeficiency virus (HIV) infection is the most attractive. The envelope glycoprotein-120 (gp-120) is the most studied target, and the attempt to use T cells targeting gp-120 to treat HIV infection started as early as 1991.^112^ Currently, promising results have been achieved in anti-HIV infection CAR T therapy.^113–115^ In addition, HIV infection of CD4-positive T cells has been well solved by gene editing methods.^115^ The HIV virus is unstable and can easily escape single-target treatment. To solve this problem, bi-and tri-specific CAR T cells have been designed for the treatment of HIV infection.^113,114^ CAR T therapies have also been explored for other viruses, such as hepatitis B virus, hepatitis C virus, and cytomegalovirus, as well as opportunistic fungus.^116–119^ Undesired autoimmune disease is another promising application scenario of CAR T therapy.^120^ On the one hand, cytotoxic T cells can specifically eliminate B cells that cause autoimmune responses by virtue of the specificity of antibodies;^120,121^ on the other hand, regulatory T cells can be redirected to inflammation sites to suppress autoimmune responses.^122,123^

Immunotherapy has become a hotspot in cancer research, and T cell-centered immunity mechanisms have gradually been revealed. Along with advances in genetic engineering, it has become possible for us to consciously and creatively exploit the immune system to fight against cancer and other diseases. When naturally occurring T cells cannot effectively control diseases, modified T cells may be a better choice. For researchers in this field, a better understanding of the biological characteristics of T cells and the pathology of different diseases will help to design “better” T cells to function in a wide range of diseases, and this also emphasizes the importance of the cooperation among different fields.

## Supplementary information

Supplementary Table 1

Supplementary Table 2

Supplementary Table 4

Supplementary Table 3

Supplementary Table 5
