# Supplementary material for: Clinical development of CAR T cell therapy in China: 2020 update
Source: Cell Mol Immunol. 2020 Sep 30;18(4):792–804. doi: 10.1038/s41423-020-00555-x (PMC8115146; doi:10.1038/s41423-020-00555-x)
Supplement: Supplementary file 3 — Supplementary Table 4 [file 41423_2020_555_MOESM3_ESM.docx]

| PMID | Year | Title |
| --- | --- | --- |
| 16648493 | 2006 | Treatment of Metastatic Renal Cell Carcinoma With Autologous T-lymphocytes Genetically Retargeted Against Carbonic Anhydrase IX: First Clinical Experience |
| 17062687 | 2006 | A phase I study on adoptive immunotherapy using gene-modified T cells for ovarian cancer |
| 17299405 | 2007 | Adoptive transfer of chimeric antigen receptor re-directed cytolytic T lymphocyte clones in patients with neuroblastoma |
| 20179677 | 2010 | Case report of a serious adverse event following the administration of T cells transduced with a chimeric antigen receptor recognizing ERBB2 |
| 20304086 | 2010 | Antitransgene rejection responses contribute to attenuated persistence of adoptively transferred CD20/CD19-specific chimeric antigen receptor redirected T cells in humans |
| 20668228 | 2010 | Eradication of B-lineage cells and regression of lymphoma in a patient treated with autologous T cells genetically engineered to recognize CD19 |
| 21540550 | 2011 | CD28 costimulation improves expansion and persistence of chimeric antigen receptor–modified T cells in lymphoma patients |
| 21830940 | 2011 | Chimeric antigen receptor-modified T cells in chronic lymphoid leukemia |
| 21832238 | 2011 | T cells with chimeric antigen receptors have potent antitumor effects and can establish memory in patients with advanced leukemia |
| 21849486 | 2011 | Safety and persistence of adoptively transferred autologous CD19-targeted T cells in patients with relapsed or chemotherapy refractory B-cell leukemias |
| 21984804 | 2011 | Antitumor activity and long-term fate of chimeric antigen receptor-positive T cells in patients with neuroblastoma |
| 22107246 | 2012 | Infusing CD19-directed T cells to augment disease control in patients undergoing autologous hematopoietic stem-cell transplantation for advanced B-lymphoid malignancies |
| 22160384 | 2012 | B-cell depletion and remissions of malignancy along with cytokine-associated toxicity in a clinical trial of anti-CD19 chimeric-antigen-receptor-transduced T cells |
| 22308288 | 2012 | CD20-specific adoptive immunotherapy for lymphoma using a chimeric antigen receptor with both CD28 and 4-1BB domains: pilot clinical trial results |
| 22553251 | 2012 | Decade-long safety and function of retroviral-modified chimeric antigen receptor T cells |
| 22863016 | 2012 | A phase I clinical trial of adoptive transfer of folate receptor-alpha redirected autologous T cells for recurrent ovarian cancer |
| 23259649 | 2012 | Re-directed T cells for the treatment of fibroblast activation protein (FAP)-positive malignant pleural mesothelioma (FAPME-1) |
| 23388583 | 2013 | Long-term stability of T-cell activation and transduction components critical to the processing of clinical batches of gene-engineered T cells |
| 23423337 | 2013 | Treatment of metastatic renal cell carcinoma with CAIX CAR-engineered T cells: clinical evaluation and management of on-target toxicity |
| 23515080 | 2013 | CD19-targeted T cells rapidly induce molecular remissions in adults with chemotherapy-refractory acute lymphoblastic leukemia |
| 23527958 | 2013 | Chimeric Antigen Receptor–Modified T Cells for Acute Lymphoid Leukemia |
| 23831595 | 2013 | Persistence and efficacy of second generation CAR T cell against the LeY antigen in acute myeloid leukemia |
| 24030379 | 2013 | Infusion of donor-derived CD19-redirected virus-specific T cells for B-cell malignancies relapsed after allogeneic stem cell transplant: a phase 1 study |
| 24055823 | 2013 | Donor-derived CD19-targeted T cells cause regression of malignancy persisting after allogeneic hematopoietic stem cell transplantation |
| 24099518 | 2013 | Design of a phase I clinical trial to evaluate intratumoral delivery of ErbB-targeted chimeric antigen receptor T-cells in locally advanced or recurrent head and neck cancer |
| 24190544 | 2014 | Definition and application of good manufacturing process-compliant production of CEA-specific chimeric antigen receptor expressing T-cells for phase I/II clinical trial |
| 24553386 | 2014 | Efficacy and Toxicity Management of 19-28z CAR T Cell Therapy in B Cell Acute Lymphoblastic Leukemia |
| 24579088 | 2014 | Mesothelin-specific chimeric antigen receptor mRNA-engineered T cells induce anti-tumor activity in solid malignancies |
| 24628073 | 2014 | Effect of anti-CCR4 monoclonal antibody (mogamulizumab) on adult T-cell leukemia-lymphoma: cutaneous adverse reactions may predict the prognosis |
| 24777247 | 2013 | T cells expressing chimeric antigen receptors can cause anaphylaxis in humans |
| 24782509 | 2014 | Closely related T-memory stem cells correlate with in vivo expansion of CAR.CD19-T cells and are preserved by IL-7 and IL-15 |
| 25154820 | 2015 | Chemotherapy-refractory diffuse large B-cell lymphoma and indolent B-cell malignancies can be effectively treated with autologous T cells expressing an anti-CD19 chimeric antigen receptor |
| 25174587 | 2015 | Treatment of CD33-directed chimeric antigen receptor-modified T cells in one patient with relapsed and refractory acute myeloid leukemia |
| 25277132 | 2014 | Neutrophil:lymphocyte ratios and serum cytokine changes after hepatic artery chimeric antigen receptor-modified T-cell infusions for liver metastases |
| 25317870 | 2014 | Chimeric antigen receptor T cells for sustained remissions in leukemia |
| 25319501 | 2015 | T cells expressing CD19 chimeric antigen receptors for acute lymphoblastic leukaemia in children and young adults: a phase 1 dose-escalation trial |
| 25444722 | 2014 | Effective response and delayed toxicities of refractory advanced diffuse large B-cell lymphoma treated by CD20-directed chimeric antigen receptor-modified T cells |
| 25800760 | 2015 | Human Epidermal Growth Factor Receptor 2 (HER2) -Specific Chimeric Antigen Receptor-Modified T Cells for the Immunotherapy of HER2-Positive Sarcoma |
| 25850950 | 2015 | Phase I Hepatic Immunotherapy for Metastases Study of Intra-Arterial Chimeric Antigen Receptor-Modified T-cell Therapy for CEA+ Liver Metastases |
| 25887777 | 2015 | A phase 1 study of the bispecific anti-CD30/CD16A antibody construct AFM13 in patients with relapsed or refractory Hodgkin lymphoma |
| 26059190 | 2015 | Bioactivity and Safety of IL13R伪2-Redirected Chimeric Antigen Receptor CD8+ T Cells in Patients with Recurrent Glioblastoma |
| 26333935 | 2015 | Chimeric antigen receptor T cells persist and induce sustained remissions in relapsed refractory chronic lymphocytic leukemia |
| 26352815 | 2015 | Chimeric Antigen Receptor T Cells against CD19 for Multiple Myeloma |
| 26451310 | 2015 | Tolerance and efficacy of autologous or donor-derived T cells expressing CD19 chimeric antigen receptors in adult B-ALL with extramedullary leukemia |
| 26811520 | 2016 | Allogeneic T cells that express an anti-CD19 chimeric antigen receptor induce remissions of B-cell malignancies that progress after allogeneic hematopoietic stem-cell transplantation without causing graft-versus-host disease |
| 26813675 | 2016 | Ibrutinib enhances chimeric antigen receptor T-cell engraftment and efficacy in leukemia |
| 26961900 | 2016 | Phase 1 clinical trial demonstrated that MUC1 positive metastatic seminal vesicle cancer can be effectively eradicated by modified Anti-MUC1 chimeric antigen receptor transduced T cells |
| 26968708 | 2016 | Chimeric antigen receptor-modified T cells for the immunotherapy of patients with EGFR-expressing advanced relapsed/refractory non-small cell lung cancer |
| 27019998 | 2016 | GD2-specific CAR T Cells Undergo Potent Activation and Deletion Following Antigen Encounter but can be Protected From Activation-induced Cell Death by PD-1 Blockade |
| 27111235 | 2016 | CD19 CAR-T cells of defined CD4+:CD8+ composition in adult B cell ALL patients |
| 27118452 | 2016 | Phase 1 studies of central memory-derived CD19 CAR T-cell therapy following autologous HSCT in patients with B-cell NHL |
| 27166358 | 2016 | Persistence of long-lived plasma cells and humoral immunity in individuals responding to CD19-directed CAR T-cell therapy |
| 27210719 | 2016 | Myeloid cells in peripheral blood mononuclear cell concentrates inhibit the expansion of chimeric antigen receptor T cells |
| 27270177 | 2016 | Clinical responses with T lymphocytes targeting malignancy-associated κ light chains |
| 27284065 | 2016 | Treatment of metastatic renal cell carcinoma (mRCC) with CAIX CAR-engineered T-cells-a completed study overview |
| 27324746 | 2016 | Phase I Trial of Anti-PSMA Designer CAR-T Cells in Prostate Cancer: Possible Role for Interacting Interleukin 2-T Cell Pharmacodynamics as a Determinant of Clinical Response |
| 27412889 | 2016 | T cells expressing an anti-B-cell maturation antigen chimeric antigen receptor cause remissions of multiple myeloma |
| 27482888 | 2016 | Phase I trials using Sleeping Beauty to generate CD19-specific CAR T cells |
| 27526682 | 2016 | Predominant cerebral cytokine release syndrome in CD19-directed chimeric antigen receptor-modified T cell therapy |
| 27582488 | 2017 | Autologous T cells expressing CD30 chimeric antigen receptors for relapsed or refractory Hodgkin’s lymphoma: an open-label phase I trial |
| 27605551 | 2016 | Immunotherapy of non-Hodgkin's lymphoma with a defined ratio of CD8+ and CD4+ CD19-specific chimeric antigen receptor-modified T cells |
| 27632680 | 2017 | Cytokine Release Syndrome After Chimeric Antigen Receptor T Cell Therapy for Acute Lymphoblastic Leukemia |
| 27887660 | 2016 | Co-infusion of haplo-identical CD19-chimeric antigen receptor T cells and stem cells achieved full donor engraftment in refractory acute lymphoblastic leukemia |
| 28029927 | 2016 | Regression of Glioblastoma after Chimeric Antigen Receptor T-Cell Therapy |
| 28031179 | 2017 | PD-1 Blockade Modulates Chimeric Antigen Receptor (CAR)-modified T Cells: Refueling the CAR |
| 28039267 | 2017 | Potent Anti-leukemia Activities of Chimeric Antigen Receptor-Modified T Cells against CD19 in Chinese Patients with Relapsed/Refractory Acute Lymphocytic Leukemia |
| 28057014 | 2017 | Cocktail treatment with EGFR-specific and CD133-specific chimeric antigen receptor-modified T cells in a patient with advanced cholangiocarcinoma |
| 28100832 | 2017 | Reporter gene imaging of targeted T cell immunotherapy in recurrent glioma |
| 28123068 | 2017 | Molecular remission of infant B-ALL after infusion of universal TALEN gene-edited CAR T cells |
| 28126984 | 2017 | Vaccination to improve the persistence of CD19CAR gene-modified T cells in relapsed pediatric acute lymphoblastic leukemia |
| 28129122 | 2017 | Phase 1 Results of ZUMA-1: A Multicenter Study of KTE-C19 Anti-CD19 CAR T Cell Therapy in Refractory Aggressive Lymphoma |
| 28183713 | 2017 | Vaccination Targeting Native Receptors to Enhance the Function and Proliferation of Chimeric Antigen Receptor (CAR)-Modified T Cells |
| 28204981 | 2017 | Phase 1b trial of proteasome inhibitor carfilzomib with irinotecan in lung cancer and other irinotecan-sensitive malignancies that have progressed on prior therapy (Onyx IST reference number: CAR-IST-553) |
| 28344808 | 2017 | Safety, tumor trafficking and immunogenicity of chimeric antigen receptor (CAR)-T cells specific for TAG-72 in colorectal cancer |
| 28366766 | 2017 | Phase I Escalating-Dose Trial of CAR-T Therapy Targeting CEA(+) Metastatic Colorectal Cancers |
| 28408462 | 2017 | Intent-to-treat leukemia remission by CD19 CAR T cells of defined formulation and dose in children and young adults |
| 28426845 | 2017 | HER2-Specific Chimeric Antigen Receptor-Modified Virus-Specific T Cells for Progressive Glioblastoma: A Phase 1 Dose-Escalation Trial |
| 28577043 | 2017 | Anti-CD138 chimeric antigen receptor-modified T cell therapy for multiple myeloma with extensive extramedullary involvement |
| 28660319 | 2017 | The clinical efficacy of first-generation carcinoembryonic antigen (CEACAM5)-specific CAR T cells is limited by poor persistence and transient pre-conditioning-dependent respiratory toxicity |
| 28710747 | 2018 | Phase I study of chimeric antigen receptor modified T cells in treating HER2-positive advanced biliary tract cancers and pancreatic cancers |
| 28715249 | 2017 | Durable Molecular Remissions in Chronic Lymphocytic Leukemia Treated With CD19-Specific Chimeric Antigen Receptor-Modified T Cells After Failure of Ibrutinib |
| 28724573 | 2017 | A single dose of peripherally infused EGFRvIII-directed CAR T cells mediates antigen loss and induces adaptive resistance in patients with recurrent glioblastoma |
| 28803861 | 2017 | Long-Duration Complete Remissions of Diffuse Large B Cell Lymphoma After Anti-CD19 Chimeric Antigen Receptor T Cell Therapy |
| 28805662 | 2017 | Clinical and immunological responses after CD30-specific chimeric antigen receptor-redirected lymphocytes |
| 28834486 | 2017 | Anti-CD19 CAR T cells in CNS diffuse large-B-cell lymphoma |
| 28924019 | 2017 | Kinetics and biomarkers of severe cytokine release syndrome after CD19 chimeric antigen receptor-modified T-cell therapy |
| 29109077 | 2017 | Safety and Efficacy of Intratumoral Injections of Chimeric Antigen Receptor (CAR) T Cells in Metastatic Breast Cancer |
| 29138340 | 2017 | Phase I Study of Chimeric Antigen Receptor-Modified T Cells in Patients with EGFR-positive Advanced Biliary Tract Cancers |
| 29155426 | 2018 | CD22-targeted CAR T cells induce remission in B-ALL that is naive or resistant to CD19-targeted CAR immunotherapy |
| 29226764 | 2017 | Chimeric Antigen Receptor T Cells in Refractory B-Cell Lymphomas |
| 29226797 | 2017 | Axicabtagene Ciloleucel CAR T-Cell Therapy in Refractory Large B-Cell Lymphoma |
| 29263894 | 2016 | Treatment of CD20-directed Chimeric Antigen Receptor-modified T cells in patients with relapsed or refractory B-cell non-Hodgkin lymphoma: an early phase IIa trial report |
| 29385370 | 2018 | Tisagenlecleucel in Children and Young Adults with B-Cell Lymphoblastic Leukemia |
| 29385376 | 2018 | Long-Term Follow-up of CD19 CAR Therapy in Acute Lymphoblastic Leukemia |
| 29458388 | 2018 | A novel generation 1928zT2 CAR T cells induce remission in extramedullary relapse of acute lymphoblastic leukemia |
| 29481659 | 2018 | Cytokine Release Syndrome Grade as a Predictive Marker for Infections in Patients With Relapsed or Refractory B-Cell Acute Lymphoblastic Leukemia Treated With Chimeric Antigen Receptor T Cells |
| 29503204 | 2018 | In Vivo Expansion and Antitumor Activity of Coinfused CD28- and 4-1BB-Engineered CAR-T Cells in Patients with B Cell Leukemia |
| 29567081 | 2018 | Activity of Mesothelin-Specific Chimeric Antigen Receptor T Cells Against Pancreatic Carcinoma Metastases in a Phase 1 Trial |
| 29637550 | 2018 | Treatment of acute lymphoblastic leukaemia with the second generation of CD19 CAR-T containing either CD28 or 4-1BB |
| 29669947 | 2018 | Anti-CD19 CAR T cells with high-dose melphalan and autologous stem cell transplantation for refractory multiple myeloma |
| 29807781 | 2018 | Dominant-Negative TGF-β Receptor Enhances PSMA-Targeted Human CAR T Cell Proliferation And Augments Prostate Cancer Eradication |
| 29812997 | 2018 | T Cells Genetically Modified to Express an Anti–B-Cell Maturation Antigen Chimeric Antigen Receptor Cause Remissions of Poor-Prognosis Relapsed Multiple Myeloma |
| 29849141 | 2018 | Disruption of TET2 promotes the therapeutic efficacy of CD19-targeted T cells |
| 29880584 | 2018 | Clinical and Biological Correlates of Neurotoxicity Associated with CAR T-cell Therapy in Patients with B-cell Acute Lymphoblastic Leukemia |
| 29895668 | 2018 | Preinfusion polyfunctional anti-CD19 chimeric antigen receptor T cells are associated with clinical outcomes in NHL |
| 29900044 | 2018 | CD133-directed CAR T cells for advanced metastasis malignancies: A phase I trial |
| 29910179 | 2018 | Autologous CD19-Targeted CAR T Cells in Patients with Residual CLL following Initial Purine Analog-Based Therapy |
| 29925499 | 2018 | Nonviral RNA chimeric antigen receptor-modified T cells in patients with Hodgkin lymphoma |
| 30030295 | 2018 | Reducing Ex Vivo Culture Improves the Antileukemic Activity of Chimeric Antigen Receptor (CAR) T Cells |
| 30048343 | 2018 | Systematic Evaluation of Neurotoxicity in Children and Young Adults Undergoing CD22 Chimeric Antigen Receptor T-Cell Therapy |
| 30097433 | 2018 | A Phase I/IIa Trial Using CD19-Targeted Third-Generation CAR T Cells for Lymphoma and Leukemia |
| 30178481 | 2018 | Neurotoxicity after CTL019 in a pediatric and young adult cohort |
| 30187944 | 2018 | Locally produced CD19 CAR T cells leading to clinical remissions in medullary and extramedullary relapsed acute lymphoblastic leukemia |
| 30275568 | 2018 | Induction of resistance to chimeric antigen receptor T cell therapy by transduction of a single leukemic B cell |
| 30348186 | 2018 | Anti-BCMA CAR-T cells for treatment of plasma cell dyscrasia: case report on POEMS syndrome and multiple myeloma |
| 30396908 | 2019 | Phase I Trial of Autologous CAR T Cells Targeting NKG2D Ligands in Patients with AML/MDS and Multiple Myeloma |
| 30501490 | 2019 | Tisagenlecleucel in Adult Relapsed or Refractory Diffuse Large B-Cell Lymphoma |
| 30518502 | 2019 | Long-term safety and activity of axicabtagene ciloleucel in refractory large B-cell lymphoma (ZUMA-1): a single-arm, multicentre, phase 1-2 trial |
| 30572922 | 2018 | A phase 1, open-label study of LCAR-B38M, a chimeric antigen receptor T cell therapy directed against B cell maturation antigen, in patients with relapsed or refractory multiple myeloma |
| 30728140 | 2019 | Factors associated with durable EFS in adult B-cell ALL patients achieving MRD-negative CR after CD19 CAR T-cell therapy |
| 30782611 | 2019 | The response to lymphodepletion impacts PFS in patients with aggressive non-Hodgkin lymphoma treated with CD19 CAR T cells |
| 30860496 | 2019 | CD19 CAR T cell product and disease attributes predict leukemia remission durability |
| 30896447 | 2019 | B cell maturation antigen-specific CAR T cells are clinically active in multiple myeloma |
| 30938714 | 2019 | Safety and tolerability of conditioning chemotherapy followed by CD19-targeted CAR T cells for relapsed/refractory CLL |
| 30988175 | 2019 | Exploratory trial of a biepitopic CAR T-targeting B cell maturation antigen in relapsed/refractory multiple myeloma |
| 31011207 | 2019 | A safe and potent anti-CD19 CAR T cell therapy |
| 31042825 | 2019 | Anti-BCMA CAR T-Cell Therapy bb2121 in Relapsed or Refractory Multiple Myeloma |
| 31055613 | 2019 | Improving the safety of CAR-T cell therapy by controlling CRS-related coagulopathy |
| 31110217 | 2019 | CD22 CAR T-cell therapy in refractory or relapsed B acute lymphoblastic leukemia |
| 31262783 | 2019 | CD19 CAR T cells following autologous transplantation in poor-risk relapsed and refractory B-cell non-Hodgkin lymphoma |
| 31321805 | 2019 | Anti-CD19 chimeric antigen receptor-modified T-cell therapy bridging to allogeneic hematopoietic stem cell transplantation for relapsed/refractory B-cell acute lymphoblastic leukemia: An open-label pragmatic clinical trial |
| 31378662 | 2019 | A combination of humanised anti-CD19 and anti-BCMA CAR T cells in patients with relapsed or refractory multiple myeloma: a single-arm, phase 2 trial |
| 31420241 | 2019 | Phase I Study of Lentiviral-Transduced Chimeric Antigen Receptor-Modified T Cells Recognizing Mesothelin in Advanced Solid Cancers |
| 31465532 | 2019 | Evidence of long-lasting anti-CD19 activity of engrafted CD19 chimeric antigen receptor-modified T cells in a phase I study targeting pediatrics with acute lymphoblastic leukemia |
| 31477906 | 2019 | Enhanced CAR T cell expansion and prolonged persistence in pediatric patients with ALL treated with a low-affinity CD19 CAR |
| 31489688 | 2019 | Shortening the ex vivo culture of CD19-specific CAR T-cells retains potent efficacy against acute lymphoblastic leukemia without CAR T-cell-related encephalopathy syndrome or severe cytokine release syndrome |
| 31606419 | 2019 | Patient-reported Quality of Life After Tisagenlecleucel Infusion in Children and Young Adults With Relapsed or Refractory B-cell Acute Lymphoblastic Leukaemia: A Global, Single-Arm, Phase 2 Trial |
| 31648294 | 2019 | High rate of durable complete remission in follicular lymphoma after CD19 CAR-T cell immunotherapy |
| 31650176 | 2019 | Toxicity and response after CD19-specific CAR T-cell therapy in pediatric/young adult relapsed/refractory B-ALL |
| 31651858 | 2019 | CD56-chimeric antigen receptor T-cell therapy for refractory/recurrent rhabdomyosarcoma: A 3.5-year follow-up case report |
| 31697824 | 2020 | Efficacy and safety of CAR19/22 T-cell cocktail therapy in patients with refractory/relapsed B-cell malignancies |
| 31725148 | 2020 | Sequential CD19-22 CAR T therapy induces sustained remission in children with r/r B-ALL |
| 31924795 | 2020 | Clonal kinetics and single-cell transcriptional profiling of CAR-T cells in patients undergoing CD19 CAR-T immunotherapy |
| 31959992 | 2020 | Safety and feasibility of anti-CD19 CAR T cells with fully human binding domains in patients with B-cell lymphoma |
| 32242358 | 2020 | KTE-X19 CAR T-Cell Therapy in Relapsed or Refractory Mantle-Cell Lymphoma |
| 32321169 | 2020 | Early response observed in pediatric patients with relapsed/refractory Burkitt lymphoma treated with chimeric antigen receptor T cells |
| 32371538 | 2020 | Chimeric Antigen Receptor-Glypican-3 T-Cell Therapy for Advanced Hepatocellular Carcinoma: Results of Phase I Trials |
| 32527643 | 2020 | Anti-EGFR chimeric antigen receptor-modified T cells in metastatic pancreatic carcinoma: A phase I clinical trial |
| 32536980 | 2020 | Co-infusion of high-dose haploidentical donor cells and CD19-targeted CART cells achieves complete remission, successful donor engraftment and significant CART amplification in advanced ALL |
| 32556247 | 2020 | Optimized tandem CD19/CD20 CAR-engineered T cells in refractory/relapsed B cell lymphoma |

Supplementary Table 4. Published CAR T clinical reports all over the world. Reports from China are marked with green font.
